# Supplementary material for: Female calls promote song learning in male juvenile zebra finches
Source: Nat Commun. 2024 Oct 16;15:8938. doi: 10.1038/s41467-024-53251-z (PMC11484889; doi:10.1038/s41467-024-53251-z)
Supplement: Supplementary file 1 — Supplementary Information [file 41467_2024_53251_MOESM1_ESM.pdf]

***Supplementary Information File*:**

Female calls promote song learning in male juvenile zebra finches

Linda Bistere[1], Carlos M. Gomez-Guzman[1], Yirong Xiong[2], Daniela Vallentin[1*]

[1] Max Planck Institute for Biological Intelligence, Seewiesen 82319, Germany

[2] University of Tübingen, 72076 Tübingen, Germany

* To whom correspondence may be addressed:

Daniela Vallentin
Max Planck Institute for Biological Intelligence
Eberhard-Gwinner-Str.
82319 Seewiesen, Germany
daniela.vallentin@bi.mpg.de

Supplementary Figure 1

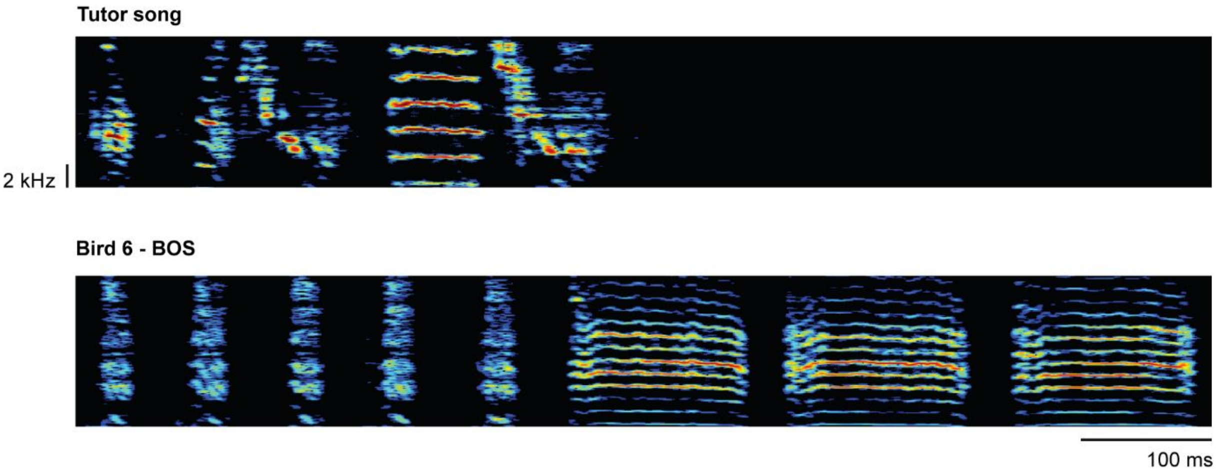

Supplementary Figure 1: Bird 6 produced a song that consisted of unmodulated distance calls. Top: Tutor song playback that bird 6 was trained with. Bottom: Birds own song from bird 6 at the last day of training (90 days post hatch). First five song elements are introductory notes.

Supplementary Figure 2

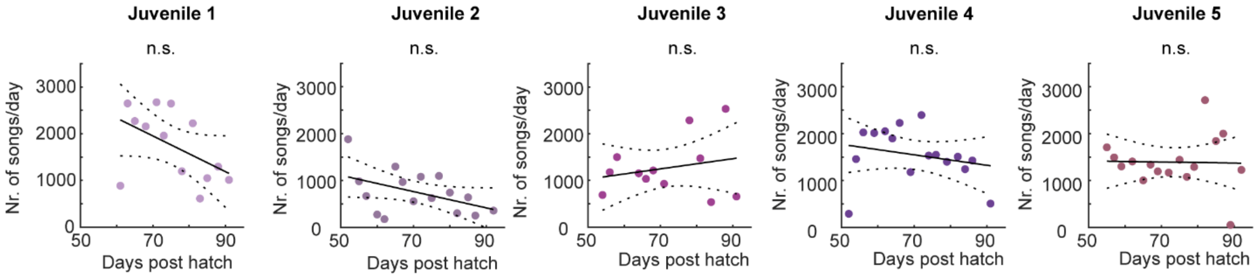

Supplementary Figure 2: Number of practice songs per day. Number of practice songs per day from 5 juveniles. Number of songs per day did not increase during the song learning phase. Source data are provided as a Source Data file.

Supplementary Figure 3

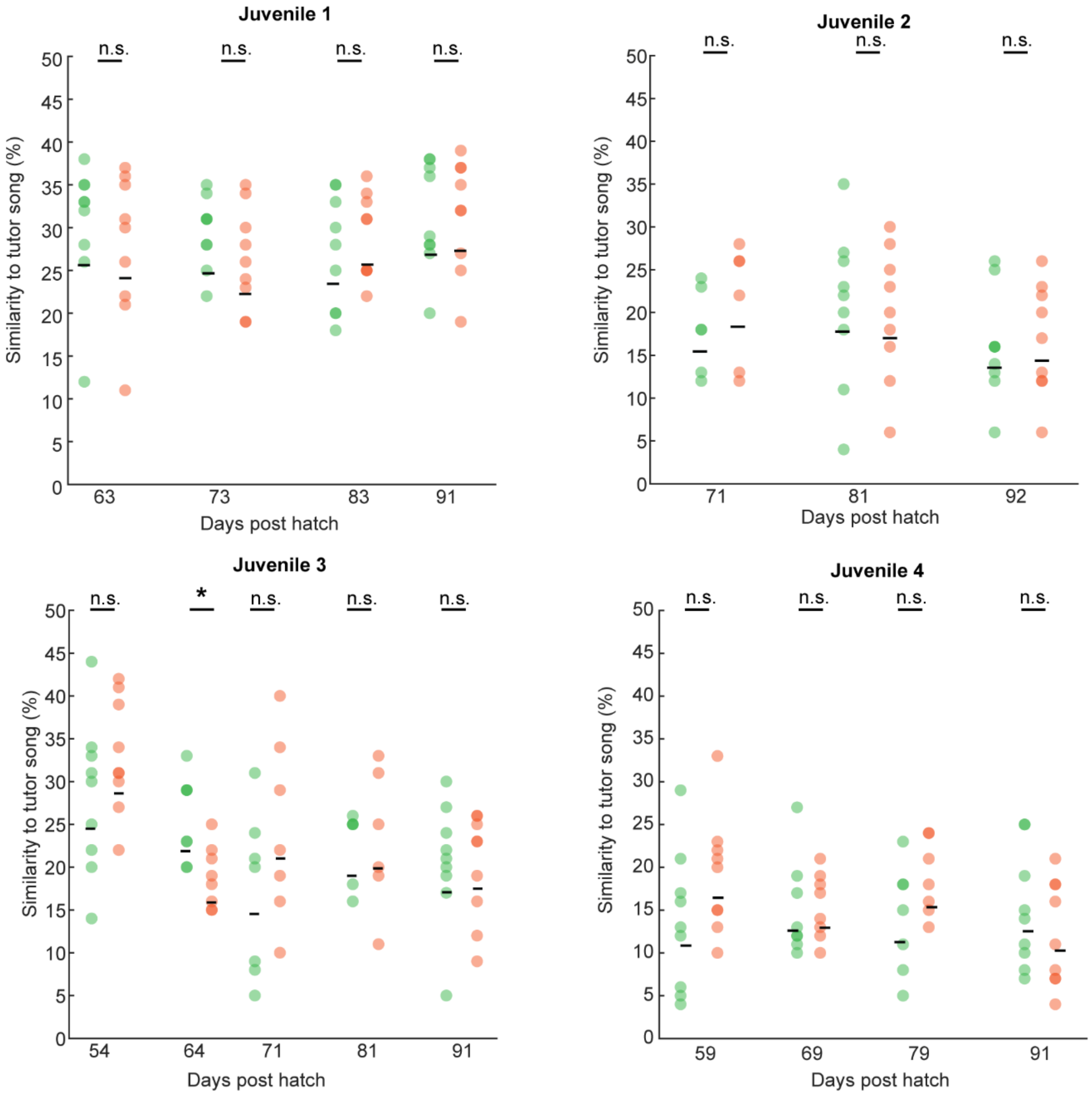

Supplementary Figure 3: Similarity of perturbed and unperturbed song to tutor song. Similarity to tutor song playback of song syllables from up to 10 songs per day that were either perturbed (orange circles) or unperturbed (green circles). Each data point represents a similarity score to tutor song of a single song snippet (either perturbed (orange) or unperturbed (green)). Juveniles 1-4 are the same birds as juveniles 1-4 in Figure 3. Reported statistic is a comparison of similarity of syllables that followed female call interruptions versus similarity of syllables that were sang without previous interruption ($p>0.05$ Wilcoxon rank sum test for all except for juvenile 3 on day 64 dph). Source data are provided as a Source Data file.

Supplementary Table 1

Genetic background or early sensory experience on song similarity after tutoring (Fig. 2)							
Number of observations	Fixed effects coefficients		Random effects coef.			Covariance parameters	
16	2		27			3	
**Formula:** Song similarity ~ 1 + social condition + (1 | father ID) + (1 | father ID:bird ID)							
**Fixed effects coefficients (95% CIs):**							
Name	Estimate	SE	tStat	DF	pValue	Lower	Upper
Intercept	-2.58815	14.508	-0.19861	14	0.84542	-33998	28.235
‘background’	27.241	8.5564	3.172	14	0.0067886	8.789	45.492

Genetic background or early sensory experience on syllable rate after tutoring (Fig. 2)							
Number of observations	Fixed effects coefficients		Random effects coef.			Covariance parameters	
16	2		27			3	
**Formula:** Syllable rate ~ 1 + social condition + (1 | father ID) + (1 | father ID:bird ID)							
**Fixed effects coefficients (95% CIs):**							
Name	Estimate	SE	tStat	DF	pValue	Lower	Upper
Intercept	2.3178	0.8617	2.6898	14	0.017605	0.46964	4.166
‘background’	2.856	0.59112	4.8315	14	0.00026635	1.5882	4.1238

Female calls on firing rate of juvenile HVC projection neurons (Fig. 4C)							
Number of observations	Fixed effects coefficients		Random effects coef.			Covariance parameters	
46	2		32			3	
**Formula:** Firing rate ~ 1 + condition + (1 | bird ID) + (1 | bird ID:cell ID)							
**Fixed effects coefficients (95% CIs):**							
Name	Estimate	SE	tStat	DF	pValue	Lower	Upper
Intercept	0.91212	2.4868	0.366786	44	0.715544	-4.0997	5.924
‘condition’	3.0066	1.3282	2.2638	44	0.028581	0.32974	5.6835

Female calls on spiking precision of juvenile HVC projection neurons (Fig. 4D)							
Number of observations	Fixed effects coefficients		Random effects coef.			Covariance parameters	
46	2		32			3	
**Formula:** Spiking precision ~ 1 + condition + (1 | bird ID) + (1 | bird ID:cell ID)							
**Fixed effects coefficients (95% CIs):**							
Name	Estimate	SE	tStat	DF	pValue	Lower	Upper
Intercept	-0.78957	0.41848	-1.8867	44	0.065805	-1.633	0.05383
‘condition’	0.78671	0.26467	2.9724	44	0.00477761	0.25329	1.3201

Female calls on subthreshold cross correlation of juvenile HVC projection neurons (Fig. 4E)							
Number of observations	Fixed effects coefficients		Random effects coef.			Covariance parameters	
46	2		32			3	
**Formula:** Subthreshold cross correlation ~ 1 + condition + (1 | bird ID) + (1 | bird ID:cell ID)							
**Fixed effects coefficients (95% CIs):**							
Name	Estimate	SE	tStat	DF	pValue	Lower	Upper
Intercept	-0.14371	0.050165	-2.8646	44	0.006376	-0.24481	-0.042604
‘condition’	0.1545	0.030224	5.1116	44	6.6629e-06	0.093583	0.21541

Difference (Δ) of subthreshold activity between silence and female call playbacks (Fig. 4F)							
Number of observations	Fixed effects coefficients		Random effects coef.			Covariance parameters	
23	1		32			3	
**Formula:** Δ Subthreshold activity ~ 1 + (1 | bird_ID) + (1 | bird_ID:cell_ID)							
**Fixed effects coefficients (95% CIs):**							
Name	Estimate	SE	tStat	DF	pValue	Lower	Upper
Intercept	0.97222	0.6678	1.4558	22	0.15956	-0.41272	2.3572

Female calls during BOS playback on firing rate of juvenile HVC projection neurons (Fig. 4H)							
Number of observations	Fixed effects coefficients		Random effects coef.			Covariance parameters	
30	2		21			3	
**Formula:** Firing rate ~ 1 + condition + (1 | bird ID) + (1 | bird ID:cell ID)							
**Fixed effects coefficients (95% CIs):**							
Name	Estimate	SE	tStat	DF	pValue	Lower	Upper
Intercept	0.70166	1.9121	0.36695	28	0.71642	-3.2152	4.6185
'condition'	2.3585	1.0507	2.2447	28	0.032873	0.20611	4.5107

Female calls during BOS playback on spiking precision of juvenile HVC projection neurons (Fig. 4I)							
Number of observations	Fixed effects coefficients		Random effects coef.			Covariance parameters	
30	2		21			3	
**Formula:** Spiking precision ~ 1 + condition + (1 | bird ID) + (1 | bird ID:cell ID)							
**Fixed effects coefficients (95% CIs):**							
Name	Estimate	SE	tStat	DF	pValue	Lower	Upper
Intercept	-0.022392	0.57952	-0.03864	28	0.96945	-1.2095	1.1647
'condition'	0.4088	0.31538	1.2962	28	0.20548	-0.23722	1.0548

Female calls during BOS playback on subthreshold cross correlation of juvenile HVC projection neurons (Fig. 4K)							
Number of observations	Fixed effects coefficients		Random effects coef.			Covariance parameters	
30	2		21			3	
**Formula:** Subthreshold cross correlation ~ 1 + condition + (1 | bird ID) + (1 | bird ID:cell ID)							
**Fixed effects coefficients (95% CIs):**							
Name	Estimate	SE	tStat	DF	pValue	Lower	Upper
Intercept	0.052106	0.062287	0.83644	28	0.40993	-0.075482	0.17969
'condition'	0.032602	0.038556	0.84557	28	0.40496	-0.046377	0.11158

Difference (Δ) in subthreshold activity between BOS and BOS with female call (Fig. 4L)							
Number of observations	Fixed effects coefficients		Random effects coef.			Covariance parameters	
15	1		21			3	
**Formula:** Δ Subthreshold activity ~ 1 + (1 | bird_ID) + (1 | bird_ID:cell_ID)							
**Fixed effects coefficients (95% CIs):**							
Name	Estimate	SE	tStat	DF	pValue	Lower	Upper
Intercept	-0.071024	1.0215	-0.06953	14	0.94555	-2.2618	2.1198

**Female calls on firing rate of adult HVC projection neurons (Fig. 5)**							
Number of observations		Fixed effects coefficients		Random effects coef.		Covariance parameters	
266		2		138		3	
**Formula:** Firing rate ~ 1 + condition + (1 | bird ID) + (1 | bird ID:cell ID)							
**Fixed effects coefficients (95% CIs):**							
Name	Estimate	SE	tStat	DF	pValue	Lower	Upper
Intercept	0.11771	0.58634	0.20076	264	0.84104	-1.0368	1.2722
‘condition’	1.0067	0.30861	3.262	264	0.001252	0.39903	1.6143

**Female calls on spiking precision of adult HVC projection neurons (Fig. 5)**							
Number of observations		Fixed effects coefficients		Random effects coef.		Covariance parameters	
248		2		129		3	
**Formula:** Spiking precision ~ 1 + condition + (1 | bird ID) + (1 | bird ID:cell ID)							
**Fixed effects coefficients (95% CIs):**							
Name	Estimate	SE	tStat	DF	pValue	Lower	Upper
Intercept	-2.5711	0.45859	-5.6065	246	5.5301e-08	-3.4744	-1.6678
‘condition’	2.4515	0.22312	10.988	246	4.1148e-23	2.0121	2.891

**Female calls on spike count of juvenile HVC projection neurons (Figure 6)**							
Number of observations		Fixed effects coefficients		Random effects coef.		Covariance parameters	
44		2		13		3	
**Formula:** Spike count ~ 1 + condition + (1 | bird ID) + (1 | bird ID:cell ID)							
**Fixed effects coefficients (95% CIs):**							
Name	Estimate	SE	tStat	DF	pValue	Lower	Upper
Intercept	0.83731	0.67152	1.2469	42	0.21934	-0.51786	2.1925
‘condition’	0.041667	0.39768	0.10477	42	0.91705	-0.76088	0.84421

Supplementary Table 1 legend: Linear mixed model results for corresponding Figures indicated in the title of each supanel.
